# CILP-2 expression in the intervertebral discs of patients with lumbar radiculopathy

**DOI:** 10.1186/s12891-024-07996-9

**Published:** 2024-11-06

**Authors:** K. Kõiv, M. Aunapuu, T. Torga, T. Rätsep, K. Bakhoff, A. Arend

**Affiliations:** 1 Department of Neurosurgery, North Estonian Medical Centre, J. Sütiste tee 19, Tallinn, 13419 Estonia; 2 Department of Anatomy, Institute of Biomedicine and Translational Medicine, University of Tartu, Ravila 19, Tartu, 50411 Estonia; 3 Department of Neurosurgery, Tartu University Hospital, Tartu, Estonia; 4 Department of Radiology, North Estonian Medical Centre, Tallinn, Estonia

**Keywords:** Intervertebral disc degeneration, Cartilage intermediate layer protein, Immunohistochemistry, Radiculopathy

## Abstract

**Background:**

Intervertebral disc (IVD) degeneration (IVDD) is one of the main causes of low back pain. One of the most important features of IVDD is the loss of extracellular matrix (ECM) with its structural components. Cartilage intermediate layer proteins (CILPs), minor glycoproteins residing in ECM, have been found to be increased in IVD as degeneration and aging progresses. The aim of the present study was to evaluate the expression of CILP-2 in the IVD of patients with lumbar radiculopathy.

**Methods:**

The IVD samples were collected from 25 patients during spinal surgery (interlaminectomy, herniated disc removal). The control IVD samples were obtained from nine patients who underwent lateral corpectomies in the thoracic region. CILP-2 expression was detected by immunohistochemistry. The patients were divided into two groups – aged under or over 50 years. A standardized clinical examination with assessment of radicular signs and deficits was performed. Subjective disability and pain were assessed using the visual analogue scale and Oswestry Disability Index (ODI). The pre-operative MRI was graded for the degree of IVD degeneration by Pfirrmann grading system. IVD samples obtained during operations were subjected to the standardized histopathological analysis applying modified Boos classification. The data were analysed by *t*-test, Mann-Whitney *U*-test, and Spearman correlation test.

**Results:**

Both histopathology scores and Pfirrmann grades did not differ between patients’ groups. Also, no correlations were found between histopathology and Pfirrmann grades, neither were any differences seen when correlating both grades to ODI, back pain or leg pain scores. CILP-2 staining was noted in all studied samples, notably strong staining was seen around large cell clusters. However, no differences in CILP-2 staining were seen between the age groups of patients. No correlations were found between CILP-2 staining and Pfirrmann grades. Grading of CILP-2 immunostaining in nine control patient samples resulted in significantly lower values. The difference is statistically significant (*P* = 0.002) compared to CILP-2 staining scores of all 25 patients’ samples.

**Conclusion:**

In this study, we detected increased CILP-2 expression in the human IVD as compared to the control group patients. CILP-2 can be a possible IVDD marker; however, as knowledge about the role of CILP-2 is limited, further studies are required.

## Introduction

Low back pain can be very frustrating and wearisome. About 80–90% of people feel annoying and disruptive discomfort in their lower spines during their lifetimes [1]. The global lifetime prevalence of low back pain is about 40% [2], and it is considered the most common health problem for which patients consult primary care clinicians [3].

Intervertebral disc (IVD) degeneration (IVDD) is one of the main causes of low back pain. IVDD may result in lumbar disc herniation accompanied by radicular leg pain, but it may also result in loss of disc height which may accelerate the degeneration of facet-joints or other paravertebral structures like ligaments and muscles, ultimately affecting the sagittal balance of the spine. All the above-mentioned structures – IVD, nerve root, facet-joints, ligaments and muscles – can also be the source of low back pain.

However, the question arises why for some people IVDD becomes more prominent than others. The aetiology of IVDD is complex and multifactorial including aging, physical exertion, trauma, smoking and genetic factors [4] mainly affecting the disc’s nucleus pulposus (NP) and its extracellular matrix (ECM).

The loss of ECM with its structural components is one of the most important features of IVD degeneration. Degradation of these components, in particular, aggrecans and collagen II, is considered a traditional sign of IVDD. However, other minor proteins residing in ECM have attained attention in IVDD studies. Namely, cartilage intermediate layer protein (CILP) has been found to be increased in IVD as degeneration and aging progresses, opposite to the decreased aggrecans and collagen II [5].

CILP is a monomeric glycoprotein originally detected in human articular cartilage in the intermediate zone. It has been shown to be implicated in several cartilage degenerative diseases including osteoarthritis [6] and IVDD [7]. Nowadays, two CILP isoforms, CILP-1 and CILP-2, have been characterised [8]. Besides cartilages, these isoforms have also been shown to be expressed in extra-skeletal tissues [6]. The two CILP isoforms are highly homologous with about 50% of the structure being similar [8]. Differences in the presence of these molecules have been associated with the progression of cartilage damage during osteoarthritis and IVDD, but some results are contradictory. For example, it has been shown in animal studies that CILP-1 and CILP-2 display different expression patterns in cartilage during osteoarthritis, with CILP-1 being upregulated and CILP-2 downregulated [6], while proteomic analysis of cartilage samples taken from patients with osteoarthritis has shown increased levels of CILP-2 [9]. Since CILP-2 rather than CILP-1 has been suggested to be related to the progression of osteoarthritis, some diagnostic value has been attributed to it [6]. In human IVD, limited information is available on the presence of CILP isoforms. Yee et al. [10] have demonstrated both CILP isoforms in the proteome of normal IVDs with decrease in samples from older individuals. However, in IVDD samples, an increase of CILP-1 and CILP-2 has been recorded [10]. Other studies employing human NP cells have shown that CILP is regulated by mechanical stress and that its expression has a negative effect on ECM synthesis [7, 11]. In transgenic mice, CILP overexpression has been shown to cause decreased signal intensity of IVDs on magnetic resonance imaging (MRI) representing an early sign of degeneration, which has further been confirmed by histological analyses detecting loss of proteoglycans and enhanced degeneration [7].

IVDD has a strong familial predisposition as a positive family history increases the risk of lumbar disc herniation [12]. MRI studies of identical twins have confirmed the genetic contribution to IVDD of lumbar spine [13]. Moreover, CILP polymorphisms have been associated with IVDD; it has been reported that CILP I395T allele is a risk factor for IVDD in Japanese male judo athletes [14], and that CILP 11,847/C is an independent genetic risk factor for lumbar IVDD in male but not female Japanese collegiate athletes [15, 16]. On the contrary, a study by Kelempisioti et al. [17] determined association between CILP polymorphism and IVDD only in females. Nevertheless, there are studies where no associations between CILP and IVDD have been found [18]. These studies demonstrate that CILP is among the molecules implicated in IVDD, and that CILP can serve as a biomarker.

Given the contradictory results of CILP content in IVDs, we aimed in this study to detect CILP-2 expression in IVDDs as compared to normal IVDs. We demonstrated higher CILP-2 staining in IVDDs compared to normal IVDs. However, no significant difference was seen in CILP-2 expression between patients’ groups aged either younger or older than 50 years.

## Materials and methods

### Patients

The lumbar IVD samples were collected at the Neurology Clinic of Tartu University Hospital (Tartu, Estonia) and the Neurosurgery Clinic of the North Estonia Medical Centre (Tallinn, Estonia) during spinal surgery from 25 patients (13 males, aged 22–81 and 12 females, aged 22–83 years). The level of IVDD was L5-S1 (9 patients), L4-L5 (13), L3-L4 (1), L2-L3 (1) and L1-L2 (1). The patients were divided into two age groups designated as GI (younger than 50 years, 7 male and 6 female patients) and GII (older than 50 years, 6 male and 6 female patients). Control IVD samples were obtained from nine patients who underwent lateral corpectomies in the thoracic region - Th12-L1 (3 patients), Th11-Th12 (3) and Th8-Th9 (1), in two cases samples were obtained from the upper thoracic region (Th1-Th2 and C7-Th1). The control group patients had no obvious degenerative disc disease. The age of seven male control patients was 33, 44, 47, 49, 50, 52 and 65 years and the age of two female control patients was 31 and 42 years. The study was approved by the Ethical Committee of Human Research, University of Tartu, in accordance with the Declaration of Helsinki (1975). Informed written consent was obtained from all patients before surgery, and they were asked to complete a questionnaire containing extra information about them (age, weight, height, smoking, working, and exercise habits). The patients were also requested to fill the Oswestry Disability Index questionnaire (to estimate ODI, the Oswestry Disability Index) and to subjectively evaluate the radicular pain in their back and leg separately on a pain-scale from 1 to 10.

### Radiology

Patients underwent MRI before surgery. MRI was performed using 1.5 and 3T magnetic resonance imaging equipment: Siemens (Avanto, Skyra, Germany), Philips (Ingenia, Nederland) or GE (Signa, Discovery, USA). To evaluate disc degeneration radiologically, modified Pfirrmann grading system was used with grades ranging from 0 to 8 [19].

### Histology

IVD samples were fixed in 10% buffered formalin solution, dehydrated and embedded into paraffin by Vacuum Infiltration Processor (Tissue-Tek^®^ VIPTM 5 Jr, Sakura, Sakura Finetek Europe B. V., Zoeterwoude, the Netherlands) according to the standard protocol. Specimens were cut with microtome Ergostar HM 200 (Microm, Germany) at four-µm thick serial sections. Tissue sections were dewaxed and stained using the hematoxylin-eosin (H&E), safranin O (Sigma-Aldrich GmbH, Germany) and alcian blue (Gee Lawson, London, England) – PAS (Sigma-Aldrich GmbH, Spain) methods for examination by light microscopy. Modified Boos classification [20] was applied with three parameters studied: (1) cell density (chondrocyte proliferation), (2) structural alterations (tears and clefts), (3) mucoid degeneration. Proliferation parameter was scored from 0 to 5 (0 = no proliferation; 1 = increased cell density; 2 = connection of two chondrocytes; 3 = small size clones, 3–7 cells; 4 = moderate size clones, 8–15 cells; 5 = huge clones, ˃15 cells); structural alterations (tears and clefts) from 0 to 4 (0 = absent; 1 = rarely present; 2 = present in intermediate amounts between 1 and 3; 3 = abundantly present; 4 = scar/tissue defects); mucoid degeneration from 0 to 3 (0 = absent; 1 = rarely present; 2 = present in intermediate amounts between 1 and 3; 3 = abundantly present). The overall histopathology grade was achieved by summing the scores of the three studied parameters; the maximum possible grade was 12. Two independent observers performed the evaluation in a blinded fashion; in the case of discrepancies, the specimens were reviewed together to formulate the final assessment. The slides were photographed with a Zeiss Axiophot 2 microscope (Zeiss, Germany).

### Immunohistochemistry (IHC)

Paraffin-embedded specimens were cut with the microtome Ergostar HM200 (Ergostar HM200, Microm, Germany) at three-µm thickness sections and mounted on AutoFrost slides (CANCER Diagnostics, Inc, USA). Then, the sections were deparaffinized in xylene and rehydrated. To ensure antibody penetration, the sections were treated with 30 µg/ml Proteinase K (Invitrogen, USA) in 50 mM Tris, pH 6.0, 5 mM CaCl2 at 37 °C for 90 min and with 0.4% bovine hyaluronidase (Fertipro, Belgium) for 3 h. Peroxidase activity was blocked by 0.6% hydrogen peroxide (Fluka, France) for 15 min followed by rinsing in PBS and blocking in Dako Antibody Diluent S2022; thereafter, the sections were incubated overnight at 4 °C with the primary CILP-2 antibody (Atlas Antibody, HPA041847, Sweden) in dilution of 1:100. Antibody was diluted in Dako Antibody Diluent (Dako Denmark A/S, Denmark). On the next day, the sections were washed in PBS and incubated with biotinylated secondary antibody (REALTM EnVisionTM Detection System, Dako Denmark A/S, Denmark) for 1 h at room temperature. The sections were washed with PBS and incubated in 3,3′ diaminobenzidine tetrahydrochloride (DAB) solution (REALTM EnVisionTM Detection System, Dako Denmark A/S, Denmark) for 10 min at room temperature in the dark, rinsed in PBS and counterstained with toluidine blue (Applichem, Darmstadt, Germany). Immunohistochemistry negative controls were performed by omitting the primary antibody.

The immunohistochemistry tissue slides were digitized using an Olympus BX-50 microscope (Olympus, Japan) equipped with Basler Ace camera (Basler AG, Ahrensburg, Germany). Microvisioneer mWSI software (Microvisioneer, Wasserburg am Inn, Germany) was used to digitise the whole section with the magnification of the objective 10×. The proportion of the stained tissue sample was assessed on the images with ImageJ 2.14.0 software applying the colour threshold function, as described by Crove and Yue [21] and used in our earlier publication [22]. In brief, the digitized tissue section was opened in the ImageJ (Fiji) program, and the “Colour threshold” tool was used to select the whole stained tissue section area. The selected area was analysed by applying the “Measure” tool. The colour threshold was adjusted to the maximum to remove the background signal, without removing the DAB signal area. The results of the DAB signal area in the form of pixels were entered into an MS Excel table, and eventually, the percentage of the DAB staining area was calculated.

### Statistical analysis

Descriptive variables (BMI and ODI) are presented as mean with standard deviation, and analysed using parametric tests (Student’s t-test, Pearson correlation coefficient, preceded by the application of Shapiro-Wilk test to confirm the presence of normal distribution). As the other variables (back pain, leg pain, histopathology, Pfirrmann and CILP-2 immunostaining) were not distributed normally (checked by Shapiro-Wilk test), nonparametric tests (Mann-Whitney U-test, Spearman correlation) were applied. Statistical analysis was performed using GraphPad Prism 9. The level of significance was set as *P* < 0.05.

## Results

### Patients’ characteristics, ODI and pain scores

**Fig. 1:**
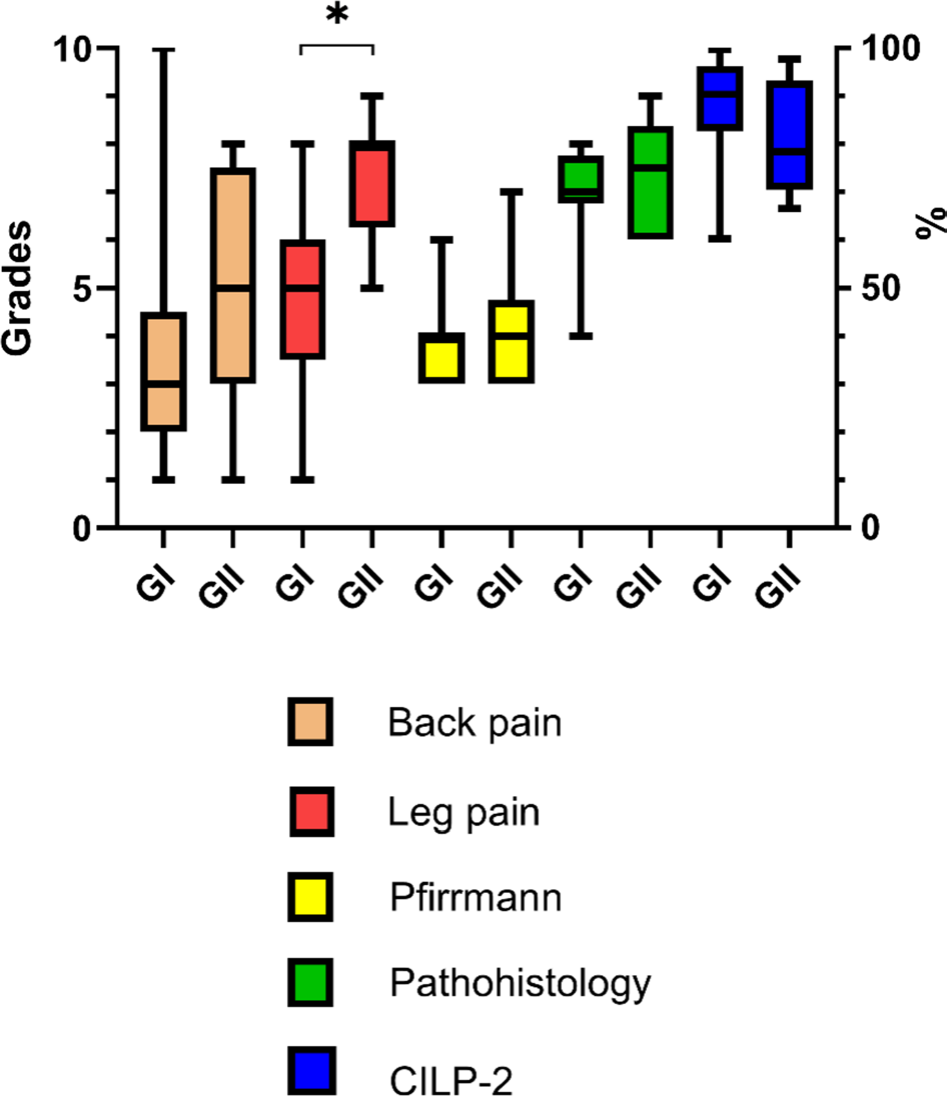
The grading of patients’ groups based on subjectively evaluated radicular pain in the back or in the legs (grading from 1–10), according to radiological disc degeneration evaluated by modified Pfirrmann grading system based on measurement of the signal intensity on T2-weighted MR images (grading ranging from 3 to 7), according to pathohistological evaluation of IVD samples (grades ranging from 0–12 based on estimations of cell density, structural alterations and mucoid degeneration) and according to the assessment of the percentage of the CILP-2 stained area. GI – group of patients younger than 50 years, GII – group of patients older than 50 years. Box-whiskers plot with 5th–95th percentiles. *P = 0.005, Mann-Whitney *U*-test

BMI demonstrated most of the patients to be overweight (BMI over 25 kg/m^2^) and, as expected, slight BMI increase was noted in the older age group (Table 1). Concerning ODI, higher average scores of back and leg pain were similarly recorded in the older age group (Table 1; Fig. 1). As compared to group I (GI), significant increase in ODI and leg pain scores was noted in group II (GII; Table 1; Fig. 1). When all patients’ data (*n* = 25) were compared, no correlations were found between BMI and ODI, BMI and back pain and BMI and leg pain. However, moderate positive correlation was found between ODI and back pain (Spearman r correlation, *r* = 0.433; *P* = 0.031) as well as ODI and leg pain (Spearman r correlation, *r* = 0.414; *P* = 0.040), while the correlation between back pain and leg pain was not significant (Spearman r correlation, *r* = 0.358; *P* = 0.079).

Noteworthy is the high proportion of smokers in the patients’ groups; in both age groups the number of smokers was higher than of non-smokers, while in the control group it was on the contrary (Table 1).

### Histopathology and Pfirrmann grades

**Fig. 2:**
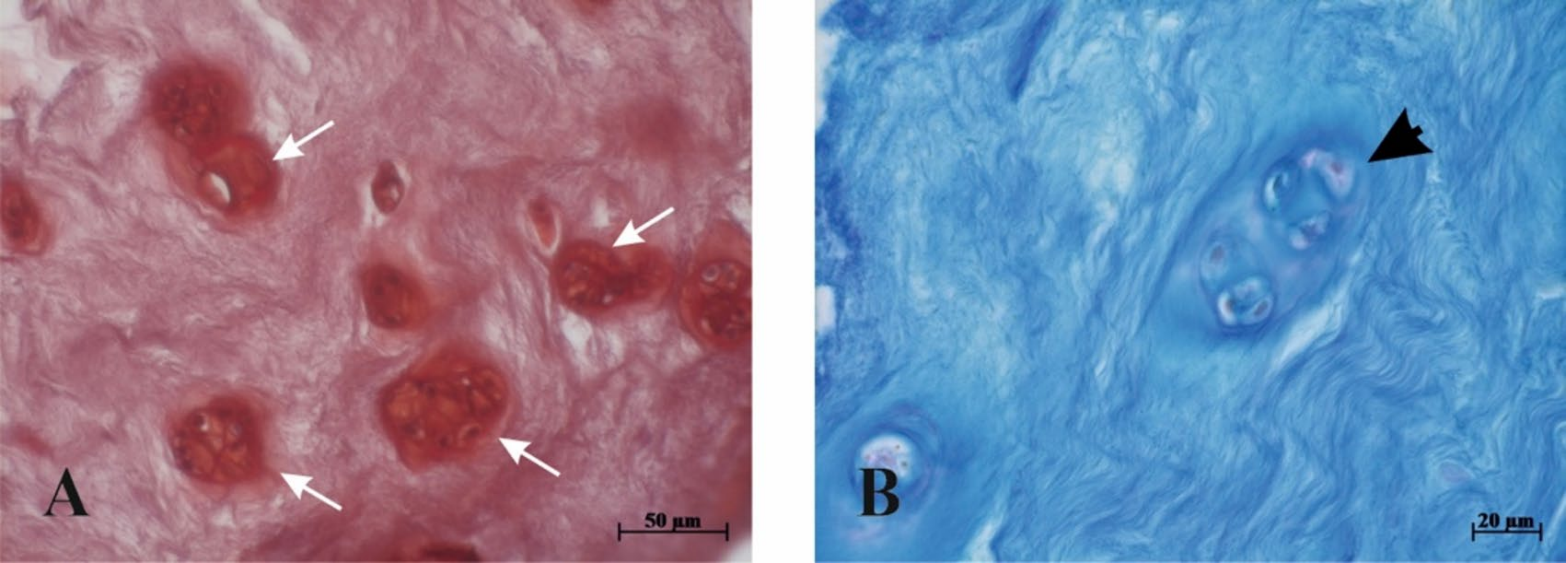
Representative micrographs of IVD samples with large clusters of cells. **A** (specimen of GI group), note large clusters of cells (white arrows), **B** (specimen of GI group), large cluster of cells surrounded by intensively stained acidic mucopolysaccharide matrix (black arrowhead). A – safranin O, B – alcian blue-PAS

Histopathology grading revealed significant damage of IVD structure. Increased proliferation of chondrocytes was seen in all prolapsed IVDs, and large clusters of cells was a constant feature of most of the samples (Fig. 2). Along with cell proliferation, the presence of fissures and mucoid degeneration contributed to the formation of the final IVD histopathology grade, which varied from 4.5 to 9 (Fig. 1). No differences were found between GI and GII. When all patients’ data (*n* = 25) were compared, no correlations were seen between histopathology grades vs. ODI, vs. back pain and vs. leg pain scores.

Concerning Pfirrmann grades, no differences were seen between the groups (Fig. 1). No correlations were found between Pfirrmann grades and ODI, back pain or leg pain scores. Also, no correlations were found between Pfirrmann grades and histopathology grades when all patients’ data (*n* = 25) were compared.

### CILP-2 immunohistochemical expression

**Fig. 3:**
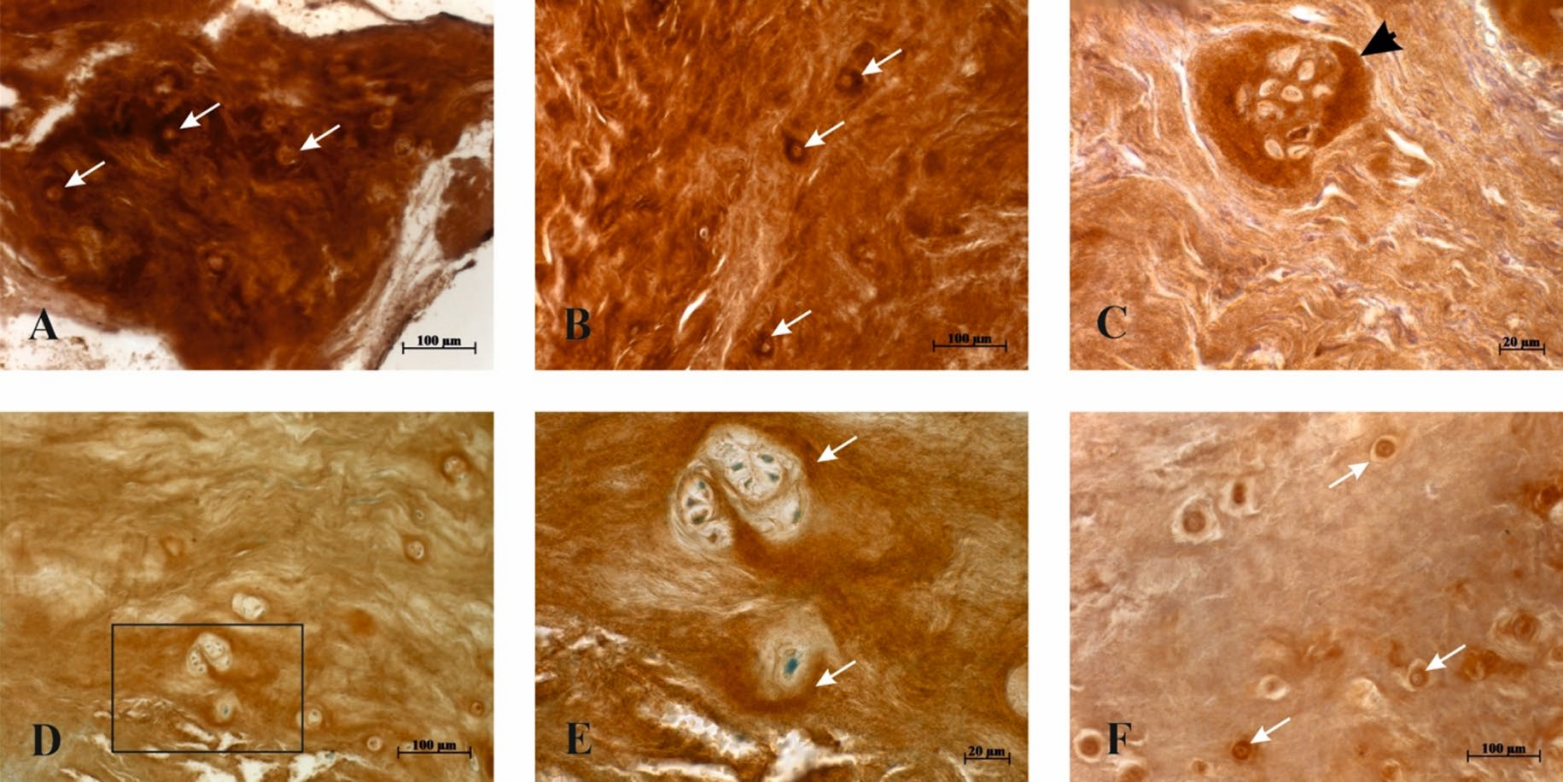
Immunohistochemical staining of CILP-2 in IVD samples. **A** (GII specimen) and **B** (GI specimen). Note CILP-2 staining in the extracellular matrix with particularly strong pericellular expression (white arrows). **C** (GI specimen). Characteristically strong CILP-2 staining around the large cluster of cells (black arrowhead). **D** and **E** (GI specimen). Note strong CILP-2 staining in the vicinity of cells (white arrows), rectangle in D presented at higher magnification in E. **F** (control group specimen). Note weak CILP-2 staining in the extracellular matrix, white arrows indicate positively stained cells. DAB + toluidine blue

CILP-2 staining was noted in all the studied samples. A characteristic feature was CILP-2 immunostaining in the near vicinity of cells, which varied from moderate to strong in both age groups. Particularly strong staining was seen around large cell clusters (Fig. 3). Estimations of CILP-2 immunostaining are given in Fig. 1, which shows no significant differences between GI and GII.

No correlations were found between CILP-2 staining estimations of all 25 patients’ samples and Pfirrmann grades (Spearman r correlation, *r*=-0.271, *P* = 0.211) or histopathology scores (Spearman r correlation, *r* = 0.032, *P* = 0.884), neither no correlations were found between the groups - Group 1: CILP-2 staining vs. Pfirrmann grades (Spearman r correlation, *r*=-0.358, *P* = 0.230) and CILP-2 staining vs. histopathology scores (Spearman r correlation, *r*=-0.358, *P* = 0.229); Group 2: CILP-2 staining vs. Pfirrmann grades (Spearman r correlation, *r* = 0.414, *P* = 0.233) and CILP-2 staining vs. histopathology scores (Spearman r correlation, *r*=-0.410, *P* = 0.248).

**Fig. 4:**
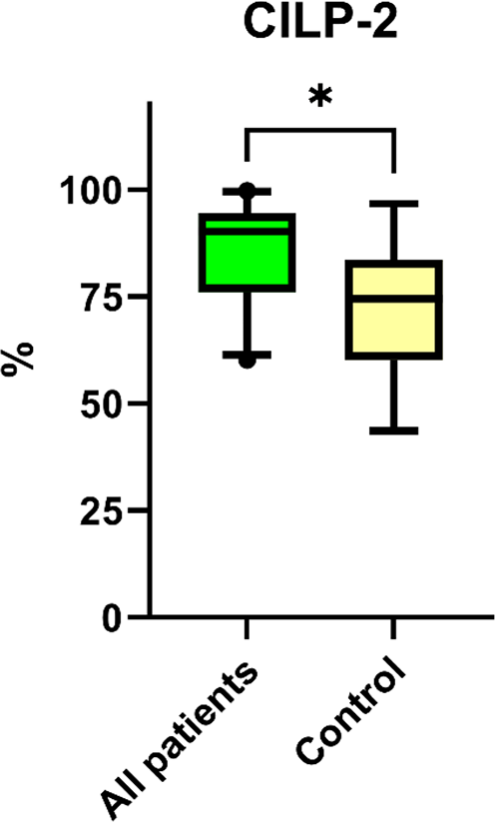
Estimations of CILP-2 immunohistochemical staining by assessing the percentage of the stained area. All lumbar discectomy patients (*n* = 25) vs. control group patients (*n* = 9), significant difference by the Mann-Whitney U-test, *P = 0.002. Box-whiskers plot with 5th–95th percentiles

However, assesment of CILP-2 immunostaining in the nine control patient samples resulted in significantly lower values, as shown in Fig. 4 and illustrated in Fig. 3F; the difference is statistically significant (*P* = 0.002) compared to CILP-2 staining scores of all the 25 patients’ samples.

**Table 1:** Patients’ characteristics and Oswestry Disability Index (ODI)

Groups (age and no of patients)	BMI	Smoking habit: yes/no	ODI
**GI** (age < 50 years; *n* = 13)	28.69 ± 8.01	9/4	22.46 ± 9.91^*^
**GII** (age > 50 years; *n* = 12)	31.04 ± 7.05	7/5	29.50 ± 6.23^*^
**Control** (mean age 47.44 ± 10.64 years, *n* = 9)	25.45 ± 5.39	3/6	-

The values are expressed as mean ± SD; *P = 0.05 GI vs. GII, Student’s *t*-test

## Discussion

The focus of our study was to detect and evaluate CILP-2 expression in the human IVD. Although CILP-1 and CILP-2 have been found by proteomic analysis to be present in the human IVD [10], this is, to the best of our knowledge, the first time CILP-2 localization has been visualized in the human IVD by immunohistochemistry. We found increased immunohistochemical staining of CILP-2 in IVDDs as compared to control group samples. Noteworthy was strong CILP-2 pericellular staining which was especially remarkable around large cell clusters. There are limited studies were CILP-1 presence in IVDDs has been confirmed by immunohistochemistry. Liu et al. [23] demonstrated increased CILP-1 immunohistochemical staining in IVDs with higher degrees of degeneration by comparing samples from patients with Pfirrmann grade III to Pfirrmann grade I. Also, Yee et al. [10] demonstrated increased CILP-1 immunohistochemical staining in IVDDs as compared to non-degenerated samples. Thus, these few immunohistochemical studies tend to support that expression of CILP-1 and, as shown by us, CILP-2 is increased in IVDDs. Concerning age, we were unable to demonstrate differences between our two study groups – no differences in CILP-2 evaluations were seen between the groups of patients aged under or over 50 years. Although in an induced rabbit disc degeneration model the expression of CILP has been found to increase with aging [5], no differences in CILP-2 were seen between the younger and older age groups in our study.

The exact function of CILP isoforms, especially in IVDs, remains poorly understood. Two CILP isoforms were originally described in 2003 by Johnson et al. [8] who used primary cultures of articular chondrocytes from the human knee. They discovered that the CILP isoforms had differential effects on chondrocyte function. In particular, CILP-1 was an IGF-1 antagonist, while CILP-2 was characterised by the lack of IGF-1 antagonism. In IVDs, CILP has been reported to bind to transforming growth factor b (TGF-b) and to prevent it from activating the transcription of cartilage matrix genes in NP cells in vitro [24]. Furthermore, it is reported that overexpression of CILP directly supresses TGF-b signalling and promotes IVD degeneration, and that CILP seems to be involved in the pathogenesis of IVD degeneration [7]. However, there is a lack of studies where the role of CILP-2 in IVDD has been specifically addressed. Studies on articular cartilages can, nevertheless, give some hints on possible CILP-2 role in the pericellular matrix. Bernardo et al. [6] showed in their ultrastructural analysis that CILP-2 was located in the human articular cartilage matrix in close proximity to collagen VI containing superstructures in chondrocytes capsular matrix suggesting potential interactions between matrix components in the territorial and inter-territorial articular cartilage matrix. In our study, we also saw characteristic CILP-2 pericellular staining; thus, we can speculate that similar interaction of CILP-2 and collagen VI may occur in IVDs. Thus, it is obvious that more detailed further studies on the role of CILP-2 in IVDD are required.

In the present study, we also analysed IVDD patients’ related data in the context of aging, i.e., between two age groups. Like with CILP-2 estimations, no differences between the two studied groups were seen, except in ODI and leg pain. Concerning patients’ characteristics, a slight but statistically insignificant tendency of BMI was noted in the older age groups, which is expected to occur concurrently with aging. If in the younger age groups four patients were of normal weight, then in the older age group only two patients were of normal weight. It is estimated that 44.8% of adults in Estonia are overweight or obese [25]; thus, our IVDD patients exceed that estimate, which further supports overweight as a risk factor in IVDD development [26]. Smoking as a known IVDD risk factor [27], was also common among our patients, as smokers outnumbered non-smokers in both age groups, on the contrary to the situation in the control group. Like with overweight, smokers among our patients exceeded the numbers expected to be present in Estonia, as the reported data show that 37.9% of men were daily and 8.8% occasional smokers in Estonia in 2010, while these numbers for women were 19% and 7.2%, respectively [28]. As reviewed by Rajesh et al. [29] several studies have found a greater number of smokers developing lumbar IVDD compared to non-smokers. Furthermore, in a recent study it was revealed for the first time that smoking is a significant risk factor causally associated with the IVDD [30], thus the detrimental effect of smoking on the health of the IVD should be taken seriously. Indeed, the sample size in our study is too small to conclusively support the role of these risk factors in IVDD. Concerning ODI, a tendency of back and leg pain parameters to increase was also seen in older age groups. However, a statistically significant difference was noted between the two age groups in ODI and leg pain, while the differences in back pain were not significant.

Degeneration assessments using MRI scans (Pfirrmann grading) and histopathology grading demonstrated no differences between the two age groups. In a larger male cadaveric study analysing the effect of aging on degeneration, moderate positive correlation has been reported with both Pfirrmann and histopathology grading [31]. In our study, however, we did not find any correlation between Pfirrmann and histopathology grades. We used a modified Pfirrmann grading system, but it has to be mentioned that, with Pfirrmann grading, changes in disc height and its signals are not linear with disc degeneration. Therefore, it is more often used with elderly patients and might not be the best suitable grading system for this case. Majeed et al. [32], in a cohort of 77 patients, did not show the quantitative histological degeneration score to correlate with Pfirrmann grading system, although they reported positive association of Pfirrmann grades with some single morphological features of IVDD, like mucoid degeneration and chondrocyte degeneration. Tertti et al. [33] similarly found no correlation between MRI findings and histopathology grading but showed MRI scanning to be sensitive to detect disc changes like decreased proteoglycan content. However, Pekala et al. [31] found moderate positive correlation between Pfirrmann and histopathology grading system in a cadaveric study; nevertheless, they concluded that Pfirrmann scale tends to deviate from the morphological assessment. Lack of correlation between MRI findings and histopathology grades in surgical cases [34] and limitation of MRI scans to correlate only with advanced stages of IVDD have also been reported [35]. It has to be stated that our study has some limitations; in particular, the study groups were relatively small to properly prove associations between patients’ data. In addition, the control group was too small to create age-matched controls to our study groups.

In summary, we demonstrated CILP-2 immunohistochemical staining in all the studied human IVD samples. As study groups were relatively small, no differences in CILP-2 staining were seen between age groups. However, increased CILP-2 expression was detected when IVDD samples were compared to the control group. Strong CILP-2 immunohistochemical staining was noted in the pericellular matrix, which was particularly evident around large cell clusters. We believe that CILP-2 can be a possible IVDD marker, but as CILP-2 functions in the IVD are still largely unknow, further studies about the role of CILP-2 in IVDD are required.

## Data Availability

The data supporting our findings can be found in the article.

## Abbreviations

IVD: Intervertebral disc
IVDD: Intervertebral disc degeneration
ECM: Extracellular matrix
CILP: Cartilage intermediate layer protein
ODI: Oswestry disability index
MRI: Magnetic resonance imaging
BMI: Body mass index
